# Participatory development of Indonesia’s national action plan for zero leprosy: strategies and interventions

**DOI:** 10.3389/fpubh.2025.1453470

**Published:** 2025-04-10

**Authors:** Perigrinus Hermin Sebong, Astri Ferdiana, Florisma Arista Riti Tegu, Deni Harbianto, Ronny Soviandhi, Asken Sinaga, Teky Budiawan, Arnoldus Janssen Angga Yanuar Risnanto, Regina Tiolina Sidjabat, Trijoko Yudopuspito, Ridwan Mawardi, Eny Setyawati, Adi Utarini

**Affiliations:** ^1^Center for Tropical Medicine, Faculty of Medicine, Public Health and Nursing, Universitas Gadjah Mada, Yogyakarta, Indonesia; ^2^Department of Public Health, Faculty of Medicine, Soegijapranata Catholic University, Semarang, Indonesia; ^3^Department of Public Health, Faculty of Medicine, Universitas Mataram, Mataram, Indonesia; ^4^NLR Indonesia, Jakarta, Indonesia; ^5^Directorate of Communicable Disease Control and Prevention, Ministry of Health, Jakarta, Indonesia; ^6^Department of Health Policy and Management, Faculty of Medicine, Public Health and Nursing, Universitas Gadjah Mada, Yogyakarta, Indonesia

**Keywords:** zero leprosy, national action plan, leprosy elimination, strategy, public health policy, disease, Indonesia, neglected tropical disease

## Abstract

**Rationale:**

Leprosy remains a significant public health problem in Indonesia, with 14,000–15,000 new cases reported each year, indicating ongoing transmission. In response to the challenges, the country needs a strategic approach to achieve zero leprosy by 2030 through creating a national action plan.

**Objective:**

To describe the development of a national action plan for leprosy in Indonesia, its strategies, and key interventions.

**Methods:**

The process of NAP-L development consisted of three phases: (1) the preparatory phase involving 78 participants in five online group discussions (OGD) and document reviews to gain an understanding of the current situation of leprosy control; (2) the implementation phase, involving eight workshops with representatives from 14 provincial health offices, six district health offices, and 78 stakeholders to discuss stakeholder mapping and key components in the national action plan; and (3) the finalization phase to produce the complete document. All workshops and OGDs were audio-recorded. Verbatim transcripts were produced from the OGDs, and a thematic qualitative analysis was carried out to identify codes and categories of barriers to leprosy control. Each workshop’s summary was documented.

**Results:**

Barriers to leprosy control were categorized into program inputs, implementation barriers from demand and supply perspectives, and proposed improvements. Four innovative strategies were formulated. The strategies were: (i) mobilizing various community resources (community); (ii) increasing the capacity of the healthcare system in the prevention, early detection, diagnosis, and management of leprosy in a comprehensive and quality manner (acceleration); (iii) improving integration and coordination with stakeholders and public-private healthcare providers (integration); and (iv) strengthening commitment, policy, and leprosy program management (commitment, policy, and management). Twenty-one key interventions and three measurable outcomes were proposed.

**Conclusion:**

The national action plan for leprosy control was developed through a participatory process involving multiple stakeholders from health and cross-sectors, public and private sectors, healthcare providers, community leaders, and persons affected by leprosy. To ensure successful implementation, a national monitoring and evaluation framework should be established to keep track of the progress and local governments should adopt the national action plan into their local health policies.

## 1 Introduction

Leprosy is a neglected tropical disease that remains a public health concern in 135 countries globally (1). Despite significant progress with the introduction of multi-drug therapy (MDT) in the 1980s, approximately 200,000 new cases are still reported annually (2). Countries such as Indonesia, India, and Brazil show persistent incidence rates, which collectively account for 74% of new cases (3, 4). Leprosy-related disabilities, particularly Grade 2 disabilities (G2D), continue to be a major concern, with delayed diagnosis and treatment leading to permanent physical and social G2D consequences (5–10). In 2022, 7,198 new G2D cases were reported globally, with a significant proportion (37.7%) reported in Southeast Asia (11). The ongoing transmission, especially among children and in areas previously considered free of leprosy highlights the need for a new strategy in leprosy control.

Long-standing challenges in leprosy control include delayed presentation of G2D cases, high proportions of multibacillary leprosy, and suboptimal treatment rates (12, 13). These issues are often due to a lack of public awareness, delays in seeking care, and insufficient healthcare capacity for early detection (14–17). Additionally, new cases in areas previously declared as leprosy-free and cases among children indicate continued transmission in the community (17–19). To address these challenges, the World Health Organization (WHO) launched the Global Leprosy Strategy (GLS) 2021–2030, which aims to achieve zero leprosy by interrupting transmission, reducing new cases, and preventing disabilities. This strategy encompasses four pillars (i.e., improving integrated active prevention and case discovery, managing leprosy and its complications, preventing new disabilities, fighting stigma, while respecting the human rights of affected individuals) and encourages each endemic country to develop a roadmap for zero leprosy relevant to its burden and country situation analysis (20, 21).

Although global initiatives such as the WHO’s Global Leprosy Strategy (GLS) 2021–2030 aim for zero leprosy transmission, many endemic countries, including Indonesia, continue to face significant challenges. The leprosy eradication program in Indonesia has started since 1932, and integrated into the primary health care system in 1969, followed by adoption of Multi-Drug combination therapy (MDT) in 1982 replacing the use of dapsone. Despite achieving national leprosy elimination status in 2000 (defined as prevalence rates of <1/10,000 population), the number of new cases were relatively high over the past 15 years (22, 23) and 98 districts-cities in six provinces remain endemic (24). Ongoing transmission and delayed diagnosis and treatment were evident. In 2022, 15,298 new leprosy cases were identified with 9.8% of which occurred in children. G2D occurred in 2.4% of children aged below 5 years (24, 25).

The continuous emergence of new leprosy cases, particularly those with G2D, and persistent challenges of leprosy control programs, coupled with the negative impact of the COVID-19 pandemic (26, 27), signify the need for Indonesia to refocus leprosy control from elimination to zero leprosy transmission in line with the GLS 2021–2030. The existing Ministry of Health regulation in 2019 that aims to achieve leprosy elimination (defined as a prevalence rate of <1/10,000 population) at the provincial level in 2019 and the district-city level by 2024 (28), and leprosy elimination at the district level as the indicator for Neglected Tropical Diseases (NTD) in the National Medium-term Development Plan 2020–2024 (23) were inadequate to respond to the challenges in Indonesia. Strategies and key interventions to overcome weak integration of services, inadequate multisectoral coordination and insufficient local government ownership were not addressed. The achievement of elimination status in some districts and cities has in fact reduced government resource allocation for leprosy control, leading to limited case-finding efforts, which have resulted in delayed diagnosis and suboptimal treatment (29). The effectiveness of the current interventions suggests a slow decline in incident cases and a substantial pool of undiagnosed cases, particularly in endemic areas. Therefore, leprosy control should go beyond a biomedical perspective and represent an inclusive concept in which the social consequences of the disease are addressed programmatically and multisectoral (30).

This has highlighted the urgent need for developing a NAP-L with a shift from merely focusing on elimination to a holistic strategy aimed at zero leprosy, addressing both medical and social dimensions of the disease. A novel approach to public health policy was used for developing the NAP-L, i.e., applying the health systems perspective and the chain for quality improvement (31). From the health system perspective, a participatory process to engage multiple stakeholders is critical toward building commitment for the implementation. In addition, the content of strategies and interventions in the NAP-L should encompass both the specific strategies for leprosy control and strategies to align with the broader transformation of the national health system relevant to leprosy. In response to the barriers in program implementation, the NAP-L considers improving the quality of program implementation, starting with the patients, families, and communities, care delivery at the microsystem level, macro systems at the organizational level, and the environment. These considerations are reflected in the rationale and structure of the action plan: (1) priority setting and interventions are emerged from a deeper understanding of the current implementation; (2) the context-related barriers (i.e., geographical access, and resource constraints); and (3) existing multisector collaboration and their activities to avoid overlapping tasks and increase sustainability. This paper describes the process of developing a national action plan for leprosy control, strategies, and key interventions.

## 2 Development of the national action plan on leprosy control

### 2.1 The core facilitators

The development of the National Action Plan for Leprosy (NAP-L) involved four main institutions: the Indonesian Ministry of Health (MoH), the Centre for Tropical Medicine, the Faculty of Medicine, Public Health and Nursing, University Gadjah Mada (UGM), NLR Indonesia, and was supported by the WHO Indonesia. The NAP-L was initiated by the MoH in collaboration with UGM and NLR as technical consultants. The MoH (RTS, ES, RM, and TY) provided direction and leadership in the Neglected Tropical Diseases program, including leprosy, while the UGM (AU, AF, and PHS) has long experience in research and technical consultancy in developing national action plans for communicable diseases such as dengue, tuberculosis, HIV/AIDS, malaria, and sexually transmitted diseases. Collaboration with NLR Indonesia (AF, AJAYR, TB, AS, JTWM), a non-government organization focusing on leprosy, enriched the capacity to facilitate the process, as they have specific, long experiences of leprosy control in close relationships with MoH Indonesia. NLR Indonesia contributed activities ranging from providing technical assistance, implementing outreach to marginal populations and people with disabilities, and advocating for policymakers.

### 2.2 Participants selection

A total of five OGDs were conducted with 78 participants consisting of representatives of the MoH, provincial health offices (PHO), and other relevant national committees (39 participants); district health offices (DHO) (6 participants); health care facilities, both the Primary Healthcare Center (PHC) and referral hospital (14 participants); academia and researchers (4 participants); and NGOs and community organizations (15 participants) (Supplementary 1). Participant selection was carried out by the core team with information primarily provided by the Ministry of Health.

The province and district health offices were selected from 14 provinces and six districts based on their level of endemicity (high and low), i.e., the number of new cases detected per 10,000 population. The participating institutions-organizations were selected purposively to represent the key stakeholders engaged and to capture those with extensive experiences in leprosy control. For instance, representatives from healthcare facilities were involved because of their roles in screenings, treatment, and detection of disability. Academia and researchers were included due to their contributions in advancing research and utilizing technology to support the leprosy control program. Non-governmental organizations (NGOs) coordinated, integrated, and collaborated with the government and private sector to eliminate leprosy, particularly in relation to disabled patients. Community-based organizations were responsible for enhancing the involvement of communities in leprosy control and collaborating with the local health facilities (Primary Health Centers) and integrated health posts or Posbindu. A detailed description of sampling criteria and justification are presented in Table 1.

**Table 1:** Roles and responsibilities of the selected participating institutions.

Ministry of Health (MoH)	Enhancing promotive and preventive efforts through policy development and technical assistance for leprosy control; Strengthening multi-sectoral actions, increasing case detection, implementing mass treatment, and improving surveillance; Strengthening the national laboratory network system, including enhancing public health laboratories for leprosy control; Engaging relevant stakeholders to secure support for leprosy control policies, particularly in eliminating stigma and discrimination and ensuring adequate financing; Assisting local governments in implementing chemoprophylaxis and supporting research and development activities
Provincial Health Office (PHO)	Implementing leprosy control programs at the provincial level; Enhancing the capacity of healthcare workers in primary and referral healthcare facilities for early detection and management of leprosy cases; Strengthening the national laboratory network system, including public health laboratories for leprosy control, and monitoring and evaluating leprosy control efforts at both primary and referral healthcare service levels
Relevant National Committee	Coordinating and synchronizing the implementation of national strategic planning and budget policies for leprosy elimination; Formulating technical guidelines for the implementation of national healthcare service guarantees and developing policies for social rehabilitation, social security, social empowerment, and social protection for leprosy patients, OYPMK (people affected by leprosy who have been cured), their families, and communities; Supporting the implementation of national-scale socialization strategies at both central and regional levels to accelerate leprosy elimination.
District Health Office (DHO)	Implementing leprosy control programs at the district/city level; Enhancing the capacity of healthcare workers in primary and referral healthcare facilities for early detection and management of leprosy cases; Coordinating with primary health centers (PHCs) to improve leprosy detection and surveillance, strengthens the national laboratory network system, including public health laboratories for leprosy control, and monitors and evaluates leprosy control efforts at primary and referral healthcare service levels.
Healthcare Facility	Treating leprosy patients and implementing leprosy control measures; Optimizing the functions of health centers, private clinics, and DPM (private medical practitioners) within essential healthcare services; Assisting community empowerment initiatives and fostering multi-stakeholder collaboration for joint action; Conducting screening and early detection of leprosy and disability cases
Academia and Researchers	Conducting research, developing innovations, and utilizing technology to support the success of the leprosy elimination program; Contributing to strengthening the capacity of healthcare workers and health cadres; Providing training in management policies, technical guidance, and the implementation of quality leprosy care services
Non-Governmental Organizations (NGOs)	Coordinating, integrating, and collaborating with the government and private sector to combat leprosy; Supporting early detection, case finding, surveillance, prevention, treatment, training, and disseminating research and development findings; Conducting community intervention to improve the lives of leprosy patients, OYPMK, and individuals with disabilities; Providing training, employment opportunities, and promoting self-care for OYPMK, and individuals with disabilities.
Community Organizations	Enhancing community participation in leprosy control activities in collaboration with village health facilities such as PHCs and Posbindu; Serving as health cadres, acting as drug adherence supervisors; Participating in health promotion activities, supporting early detection efforts, and assisting leprosy patients and OYPMK with disabilities; Community organizations are also actively involved in the Leprosy-Friendly Village program.

### 2.3 A three-phase design

**Figure 1:**
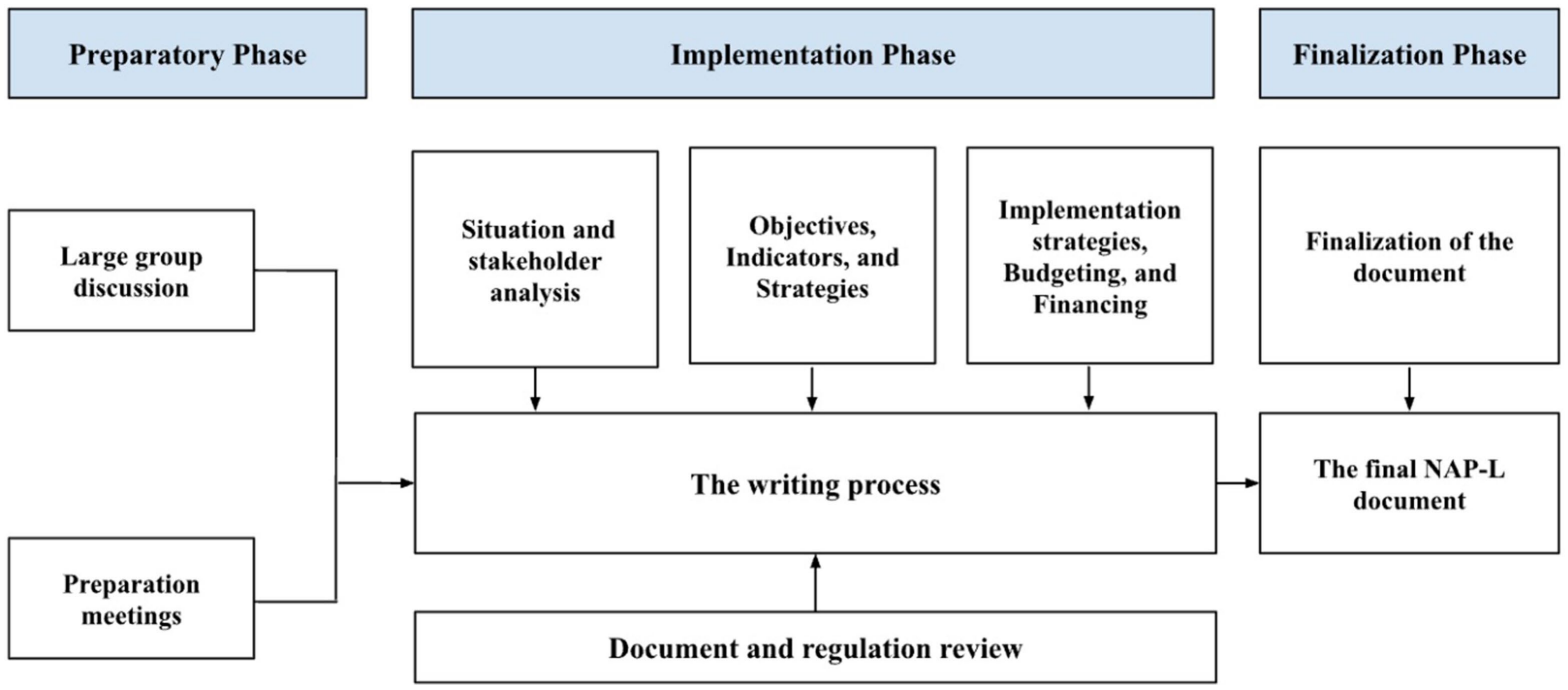
The development process of the National Action Plan for Leprosy in Indonesia, 2023–2027.

The development of NAP-L consists of three phases: preparation, implementation, and finalization (Figure 1). The preparatory phase aimed to provide an overview of Indonesia’s current leprosy control situation. Activities in this phase consisted of a kickoff meeting and desk review of the implementation phase, which aimed to discuss the content of the NAP-L. Eight workshops were organized to address the key components of the NAP-L document, consisting of situation analysis, stakeholder analysis, objectives and indicators, strategies and key interventions, implementation strategies, and program budgeting and financing. The final phase produced the NAP-L document, which was approved by the MoH.

### 2.4 The preparatory phase

Document reviews of national policies, regulations, program guidelines, and reports were conducted to summarize key information such as challenges, enablers, and best practices in leprosy control. Five OGDs were organized, and Supplementary 2 provides a detailed description of the informants’ institutions. The OGD allowed many stakeholders to interact and express their ideas, which is necessary for engaging multiple stakeholders and building a strong sense of ownership toward the NAP-L document.

All OGDs were recorded and transcribed verbatim. PHS checked the accuracy of all the transcripts. The personal information of the participants was kept confidential during transcription (i.e., I1 and I2). Thematic analysis of qualitative data was conducted following the six steps by Braun and Clarke: become familiar with the data, generate initial codes, search for themes, review themes, define themes, and write up (32). First, to become familiar with the data, the first author (PHS) read through all the transcribed OGDs several times. Verbatim transcripts were confirmed with the original audio recordings for accuracy. The co-authors (AU, AF, FART) individually read all the OGDs, with a particular emphasis to search for patterns, meanings and initial ideas for coding. PHS and FART evaluated that saturation was achieved.

Second, PHS and FART generated initial codes, and the team met to discuss and identify possible categories and themes. Third, the authors sorted the list of codes into categories. Fourth, PHS, AU, AF, FART, and other co-authors reviewed the categories and populated them into a matrix to visualize the categories and link categories. The process of identifying the themes was interactive. Fifth, codes, categories, themes, and a visual diagram were finalized to reflect the common understanding and agreement among the entire team. Finally, all categories and themes were written out in the manuscript.

### 2.5 The implementation phase

Eight workshops were conducted to discuss the following NAP-L components: (i) situation analysis, (ii) stakeholder analysis, (iii) strategic issues, (iv) proposed strategy, (v) key interventions, (vi) implementation strategies, (vii) budgeting, and (viii) financing. Each workshop involved stakeholder representatives of core institutions or organizations with a specific interest in particular roles and responsibilities in leprosy control. The detailed structure, objectives, outputs, and number of participants in each workshop are presented in Supplementary 3.

#### 2.5.1 Situation analysis and stakeholder analysis

The one-day workshop aimed to serve two objectives, notably, the epidemiological and current situation of leprosy, and identify stakeholders’ different interests, responsibilities, and roles at different levels and across multiple sectors for leprosy elimination in Indonesia. The workshop combined various methods, such as presentations on the importance of NAP-L and WHO global strategies for Leprosy and NTDs, group discussions to identify the SWOT of leprosy control programs and strategic issues, and stakeholder analysis exercises. In this exercise, a stakeholder analysis matrix, including their roles and responsibilities, was produced. Participants were asked to reflect on their roles and experiences in implementing leprosy control programs at the national and subnational levels, including stakeholders’ interests and power in the leprosy control program (stakeholder mapping).

#### 2.5.2 Strategic issues, strategies, and key interventions

The third to fifth workshops aimed to identify strategic issues or challenges and critical aspects for improvement; formulate goals, objectives, and strategies for leprosy control; and develop essential interventions and activities for each strategy. The workshops applied several methods, that is, presentation of valuable frameworks, comparison with potential strategies from the previous action plans, group work, and discussion to complete the SWOT matrix, and to reach an initial consensus regarding goals, objectives, and key strategies. Participants conducted a SWOT analysis by identifying the strengths and weaknesses of leprosy control (internal factors) and its opportunities and threats (external factors). The results of the group discussions were presented and reviewed to the large groups. This was followed by continuous discussions to reach a consensus on the goals and objectives of the NAP-L, considering the GLS 2021–2030 indicators, NTD global targets, and national baseline data for leprosy in Indonesia in 2022.

#### 2.5.3 Implementation strategies, program budgeting, and financing

The last three workshops aimed to develop implementation strategies (i.e., approaches, monitoring and evaluation mechanisms, and responsibilities), program budgeting plans, and funding sources for implementing the NAP-L. These workshops applied intensive group discussions to elaborate on the details of each key intervention (the objectives, activities, locus, regional targets, population, and service targets); described monitoring and evaluation mechanisms or monitored inputs, processes, and outputs from the NAP-L implementation; and identified key roles and responsibilities for implementing NAP-L key strategies and interventions. Participants were asked to calculate the costs required to implement the NAP-L and identify funding sources. The unit costs and volumes of each activity in the proposed key interventions were estimated to develop the budget for each strategy, followed by the identification of sources of funding by different stakeholders at different levels of government and their responsibilities for financing the program.

### 2.6 The finalization phase

The finalization phase ensured that feedback, comments, and recommendations from various stakeholders and workshop participants were received and included in the document. Two intensive workshops with core facilitators involved in developing the NAP-L document were organized to revise the draft before it was finally submitted for approval by the MoH. The writing team prepared the first draft of the NAP-L. The NAP-L document comprises six chapters. These are the introduction (Chapter 1); challenges and strategic issues (Chapter 2); objectives, indicators, and targets (Chapter 3); strategies and key interventions (Chapter 4); budgeting and financing (Chapter 5); and implementation strategies (Chapter 6).

## 3 Results

This section presents the program indicators followed by findings from the online group discussion (OGD) and workshop series to highlight the barriers to existing leprosy control and critically proposing strategies to overcome these challenges. Barriers emerged regarding program inputs and leprosy control program implementation, particularly from the demand and supply perspectives.

The situation of leprosy as a significant public health issue was worsened during the COVID-19 pandemic. Case detection rate ranged from 6.08 to 6.75 per 100,000 population in 2014–2019, and further decreased to only between 2.03 and 4.59 per 100,000 population during the COVID-19 pandemic. Likewise, the prevalence of leprosy at the national level declined from 0.69–0.79 (2014–2019) to 0.49–0.54 (2020–2022). The cumulative disability rate of 4.59 cases per 100,000 population indicates a significant burden of leprosy, as it exceeds 4 cases per 100,000. Although 81.38% of leprosy cases were detected without disabilities, the overall disability rate was still 3.6 cases per 100,000 population, with the proportion of grade 1 and grade 2 disabilities being 8.49 and 6.28%, respectively. These trends are particularly concerning among vulnerable groups, especially women and children. Among the new leprosy cases, 35.55% were women, and 10.23% were children. The proportions of G2D among women and children were 4.93 and 1.89%, respectively. These numbers, however, were likely to not capture the actual burden due to refocusing resources and programs to respond to the COVID-19 pandemic (24).

Three indicators were used in the national leprosy prevention and control, i.e., districts-cities that have achieved elimination status, the proportion of new cases without disabilities, and leprosy patients who complete the treatment timely. The latest report in 2022 stated that 390 out of 482 targeted districts-cities had eliminated leprosy (80.91%). The proportion of new leprosy cases without disabilities was 85.97%, below the 90% target. Likewise, the percentage of leprosy patients who completed their treatment timely also did not reach the 90% target (i.e., 84.97%). Delayed distribution and availability of medicine and logistics remain significant barriers to leprosy, especially the shortage of rifampicin for children and adults.

### 3.1 Barriers to the leprosy control program

**Figure 2:**
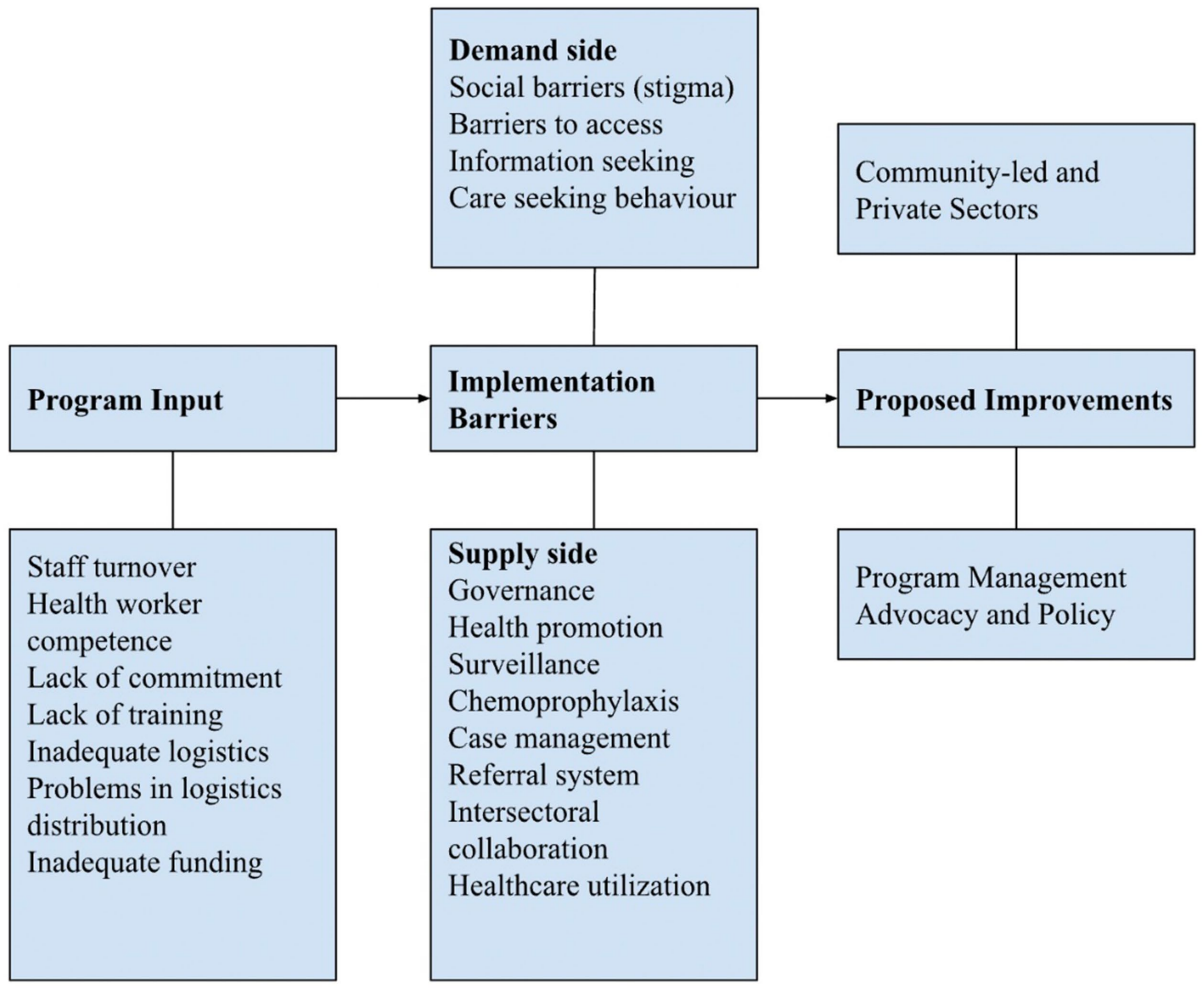
Barriers and proposed improvements in the implementation of leprosy control program.

Two major barriers to leprosy control program implementation were identified: program input and implementation. To move forward toward zero leprosy, strategies to overcome the barriers to leprosy elimination in Indonesia were proposed (Figure 2; Table 2).

**Table 2:** Categories, subcategories, and quotations from the online group discussions on leprosy control in Indonesia.

Category	Sub-category	Quotations
Program Input	Staff turnover	“There is always a frequent staff turnover. Sometimes the new program manager or deputy supervisor in the district changes, and training is needed” (PHO staff, West Papua Province)
	Health workers competence	“At that time, I had checked myself at the Puskesmas. There were two Puskesmas officers in Palopo City at that time, but they were not competent in diagnosing leprosy” (*Permata* staff)
	Lack of commitment	“In Maluku, it was difficult because many areas needing chemoprophylaxis were geographically difficult to reach while the local government had a limited budget. So, the main problem here was the lack of local government commitment. We can only hope for funding from the national level..”. (PHO staff, Maluku Province)
	Lack of training	“Only nine districts in West Java were trained. Maybe it is about funding. So, we only train health promotion workers in 9 districts” (PHO staff, West Java Province)
	Inadequate logistics	“One of our challenges is running out of MDT in the province while we actively detect leprosy cases. Children with leprosy were not being given MDT because MDT supplies are insufficient” (Staff of PHO, North Sulawesi Province)
	Distribution of logistics	“MDT was no longer distributed here but rerouted to other districts with leprosy cases. When MB leprosy cases suddenly appeared again, we have limited MDT for the district of Simeulue, which is located far away in the islands” (DHO staff, Aceh)
	Inadequate funding	“For financing from the POK, leprosy is not a priority, therefore, it is not optimized” (Vice Supervisor, East Java)
Implementation barriers		
Demand side	Social barriers (Stigma)	The stigma remains strong, and there is still no adequate information or education about leprosy in the community. Many people are still ostracized when a patient is diagnosed with leprosy, and they sometimes do not dare to seek treatment (General Practitioner, Cipamokolan *Puskesmas*)
	Barriers to access	“The obstacle is when we have to visit patients in distant locations because up to now, there is only one mobile Puskesmas officer who is responsible for many programs. Leprosy officers often have to pick up patients or control patients in the village themselves because patients with leprosy are usually poor. Even if they know they have to take the drugs from the Puskesmas, they often do not come. So, the officers have to visit them. There is no official vehicle available for leprosy officers” (General Practitioner, North Minahasa)
	Information seeking	“When I was first diagnosed with leprosy, I first accessed information on leprosy from the media. I experienced leprosy in 2012 when I already used social media to seek information. There was limited knowledge of what leprosy was, and the officer only provided information that leprosy is a skin disease” (NLR Indonesia)
	Care seeking behavior	“Every quarter, we see leprosy cases from nearly all areas, and patients are reluctant to seek treatment at *Puskesmas* [PHC]. When spotting occurred, they did not seek treatment at the nearby Puskesmas in their area. Instead, they prefer to go to faraway areas so that others are not aware of their condition” (General Practitioner, Kupang)
Supply-side	Governance	“In terms of implementation, we were already on track, even though the national and sub-national (province and district) governments were not in line. Support from the subnational government so far has not been as we expected” (MoH)
	Health promotion	What can be done to increase public awareness is socialization with the slogan “*yuk sadar bercak”* [beware of spots]. We invited the Puskesmas to accommodate the village level to make banners so that information on leprosy is always posted (similar to TB maternal and child health, or elderly health in the village). Therefore, leprosy is not less viral than other diseases (Program Officer, NLR Indonesia)
	Surveillance	“Our target achievements were lower, but that did not reflect the actual achievements because we were not optimum in tracking leprosy cases. The intensive case finding was not even implemented in 2 years. So, of course, the decline of new cases in 2021 was not the true picture, although the number was still actually under 1 per 10,000 population” (DHO staff, Cirebon)
	Chemoprophylaxis	“Even the chemoprophylaxis plan that we originally planned, we did not do it at all. Even though we planned for elimination, we were a bit horrified at this point because the data was not real data” (PHO staff, Maluku Province)
	Case management	“We did active case findings but were not supported by adequate logistics. This was an obstacle for us too. We found the cases, but drugs were not available for treatment” (PHO staff, North Kalimantan)
	Referral systems	“We had not started a referral system with private doctors and hospitals. But for the leprosy program, we tried out in 2021. Now, we continue to develop it so that for leprosy, it is also possible to be implemented by a network involving private doctors, clinics, and so on. But the crucial issue was no budget” (DHO staff, Bone)
	Intersectoral collaboration	“There are many activities carried out in collaboration with cross-sectors in the village such as semi-active surveillance (SAS) activities, these are village development activities” (Lappariaja Puskesmas staff)
	Healthcare utilization	“There was one case where the patient was unsure about going to the Puskesmas. In the end, he preferred to go to a specialist doctor, even though it is more expensive. He was then advised to go to a private laboratory, and finally, this patient did not go to the Puskesmas again. We do not know how the patient was treated because there was no link or procedure for patients seeking treatment at private health facilities” (Provincial Health office supervisor, East Java)
Proposed improvements	Community-led and private sectors	The preventive activities carried out at our Puskesmas are named “Bina Desa Sahabat Kusta.” It involves cross-sectors, religious leaders, community leaders, and cadres. So, it’s not surprising that in our services, patients are brought to us by religious leaders or community health workers when they see a spot of leprosy” (Airmadidi Puskesmas staff)
	Quality of program management	“We do not need to create a new network. We can use the existing network we have for TB [Tuberculosis]. We consider this as a joint network, initiated by the TB program. Even though this was only started in the current year, the results were extraordinary to accelerate the leprosy control program” (Staff of Disease Prevention and Control, DHO, Cirebon)
	Advocacy and policy	“Six pillars of health transformation that must be reflected in this national action plan so that it can be in line with the national health system transformations of the Ministry of Health, to be operationalized and structured” (Commissioner, National Disability Commission)

#### 3.1.1 Barriers to program input

The OGDs revealed that inputs for leprosy control programs at the provincial and district levels were often inadequate. High staff turnover, especially at the primary health center (PHC) level, and the lack of capacity of healthcare workers in leprosy case management in PHCs, as the frontline of leprosy control programs, were often expressed. Poor drug management, stockouts of MDT drugs, inadequate laboratory supplies for diagnosis, and problems in the distribution of medicine were among the logistical problems mentioned. Inadequate funding for operational activities and poor commitment from subnational governments were mentioned in most group discussions.

#### 3.1.2 Barriers to program implementation

Barriers to program implementation were identified from both the demand (i.e., persons affected by leprosy, NGO, and community) and supply sides (i.e., MoH, PHO, DHO, healthcare providers, healthcare workers, and leprosy-related government institutions at national and subnational levels).

The demand side was dominated by issues related to stigma from persons affected by leprosy (self-stigma), as perceived or imposed by the community and others (perceived stigma), and its implications, such as social rejection and discrimination. Adequate information about leprosy in response to the existing stigma is not available at health facilities, and even health workers still have stigmatizing attitudes toward the disease. Therefore, it is common practice for the community to resort to traditional healers or alternative medicine when leprosy is suspected owing to delayed diagnosis and treatment at formal healthcare facilities. For those with disabilities, concerns about barriers to accessing healthcare facilities were mentioned, including geographical barriers, poor access to people with disabilities, lack of community-based rehabilitation facilities, and patients refusing leprosy testing. People affected by leprosy who are already undergoing treatment often lack information on the side effects of drugs.

Several issues related to governance, health promotion, surveillance, chemoprophylaxis, case management, referral systems, intersectoral collaborations, and healthcare utilization were identified from a supply side perspective, as elaborated below.

##### 3.1.2.1 Governance

Almost all participants agreed that leprosy was not prioritized as much as it should be in Indonesia. The implementation of leprosy programs between the central and subnational levels was not aligned, and local governments, including those in endemic areas, had low commitment. The absence of an action plan for leprosy elimination indicates a low priority for this disease, especially on the subnational health development agenda. A poor understanding of the program policies and objectives by the implementing units (i.e., PHO, DHO, and healthcare facilities), organizations (i.e., leprosy-related government institutions, village communities, and private clinics), and other parties (i.e., NGO, academia, research institutions, and health professional associations) was also apparent.

##### 3.1.2.2 Health promotion

Health promotion activities for leprosy were conducted through health education, public campaigns, community involvement, and empowerment approaches (e.g., self-care groups). These approaches varied among provinces and were reported to have no effect on community awareness. Participants also declared that educational activities on leprosy were not conducted regularly, with limited availability of health information media. Existing health promotion media on leprosy was not attractive and not widely disseminated.

##### 3.1.2.3 Surveillance

Almost all the participants expressed the problem of limited resources for implementing the surveillance system at the subnational and district levels. Healthcare providers perceived the recording and reporting of leprosy data as burdensome and as often delayed or inaccurate. Many districts did not implement active case-finding, including regular contact tracing, owing to limited technical capacity and resources. Only a few districts conducted leprosy screening in schools and cross-border areas. The number of imported cases also increased in areas that previously had eliminated cases.

##### 3.1.2.4 Chemoprophylaxis

Although chemoprophylaxis with SDR-PEP was introduced as a national policy in 2019, its implementation remains limited. Contact tracing is not regularly conducted or integrated with SDR-PEP administration. Owing to self-stigma, patients with leprosy often do not disclose their status to their families, thus preventing contact tracing and chemoprophylaxis. The stock-out of the SDR-PEP is also frequently encountered in districts.

##### 3.1.2.5 Case management

Various problems have been encountered, such as recurrent leprosy infections, delayed case detection, dapsone allergy, lack of availability of MDT regimens for pediatric patients, and a high number of relapse cases. Challenges related to treatment and treatment monitoring were apparent, including various drug reactions appearing before, during, and after treatment; incomplete treatment; loss to follow-up; untreated drug reactions; poor treatment monitoring; patient mobility; and lack of family support. Most participants had high drug dropout and withdrawal rates, and post-RFT monitoring has not been optimized for patients with leprosy or pediatric patients. Limited hospital facilities, budget constraints, and priorities in managing new cases were the main barriers to rehabilitation.

##### 3.1.2.6 Referral system

The referral system ranged from the PHC level (such as Puskesmas) to secondary public hospitals. However, some patients prefer treatment at a private clinic or by a specialist. Care-seeking behaviors and social insurance policies complicate the referral system because of the absence of referral guidelines for private medical facilities. Not all public hospitals in leprosy-endemic areas are well equipped to perform leprosy testing and treatment when receiving referrals for leprosy cases. Poor linkages between PHC, private clinics, and hospitals and unclear procedures for patients seeking treatment in private clinics or hospitals have also been mentioned.

##### 3.1.2.7 Intersectoral collaboration

There have been limited or no efforts to synergize collaboration with the non-health sector or institutions in terms of stigma reduction, program planning, case findings, promotion, and disability prevention and treatment. Organizations for persons affected by leprosy are rarely involved in the planning, implementation, monitoring, and evaluation of leprosy control programs. Amidst the obstacles on the supply side, access to the facility and disability remain challenging due to stigma and lack of access to transport facilities.

##### 3.1.2.8 Healthcare utilization

Difficulties in accessing PHC facilities (i.e., Puskesmas and private clinics) and hospital facilities were mentioned because of the distance and hard-to-access transportation in remote areas. At the PHC level, Puskesmas offers services aimed at preventing disability, semi-active surveillance, active surveillance, and self-care group services. However, these services have not been implemented because of the distance, lack of staff, and poor communication between patients and the community. In addition, some patients are hesitant to seek care at PHC because of the lack of clear pathways or procedures for those seeking treatment at private health facilities.

These findings align with the SWOT analysis, which expresses four key issues: political support and policy; leprosy case management; surveillance; and stigma (Table 3).

**Table 3:** The Strength, Weaknesses, Opportunities and Threat (SWOT) analysis.

Political Support and Policy	Strengths: The central government has the authority to declare leprosy as a national priority; A sustainable funding allocation scheme, e.g., Bantuan Operasional Kesehatan (BOK) or Health Operational Fund to support primary health care activities; Implementation of a national health insurance policy; The availability of national policy and guidelines for leprosy prevention and control (Ministry of Health Regulation on Leprosy Prevention and Control, 2019). Opportunities: The central government can mandate local governments to develop a district action plan; Village funds may be allocated for leprosy prevention and control; Consistent support from health professionals, community, and religious organizations is essential.	Weaknesses: Leprosy has not been a priority for the national and local health sector; Technical guidelines for implementation are not available; Insufficient budget allocation for leprosy prevention and control at national and regional levels. Threats: Lack of political attention due to leprosy is considered a neglected disease; Geographical barriers and high patient mobility in several endemic areas; Lack of cross-sectoral contribution and synergy for the program implementation; Misperception toward criteria for leprosy elimination status.
Case Management	Strengths: Several national programs implemented at various levels of government, including villages (PIS-PK (*Program Indonesia Sehat dengan Pendekatan Keluarga*/Healthy Indonesia Program with Family Approach), PKH (*Program Keluarga Harapan* (Family Hope Program), KB (*Keluarga Berencana* (Family Planning), etc.), have existed and well functioned; Primary healthcare facilities (Puskesmas; private clinic) are well established and distributed in the community; Existing financial allocation schemes from various sources (i.e., BOK, village finds); The existence of activity financing sourced from village funds and the BOK Surveillance system for leprosy is well-established; Best practices in leprosy programs in rural and urban areas implemented by various parties; Digital transformation (SITASIA or *Sistem Informasi Tata Laksana Kusta Indonesia*/Indonesia’s Leprosy Management Information System), e-learning. Opportunities: The domestic pharmaceutical industry’s capacity to produce Multi Drug Therapy (MDT) and SDR (Single Dose Rifampicin) for leprosy; Leprosy can be included as a national health system indicator; Well-established OYPMK organizations, civil society organizations, community leaders, religious leaders, and cultural leaders; Program integration with the Ministry of Social Affairs for people with disabilities; Support from professional organizations (PERDOSKI (*Perhimpunan Dokter Spesialis Kulit dan Kelamin Indonesia*/Indonesian Society of Dermatology and Venereology)), IDI (*Ikatan Dokter Indonesia*/Indonesian Medical Association) that have branches throughout Indonesia; Active participation of NGOs and OYPMK in leprosy and disability prevention; Well-established community activities and cross-sector collaboration for leprosy prevention and control.	Weaknesses: Multi rugs therapy and rifampicin are not available due to poor distribution; The existing drug supply information system is inadequate; Lack of knowledge and skills in leprosy management by health workers; Limited coverage of health insurance for rehabilitation and assistive devices for leprosy patients and OYPMK (People Affected by Leprosy); Limited number of leprosy staff due to job rotation. Threats: High prevalence of indirect risk factors for leprosy, such as sanitation, poverty, and malnutrition; Increased risk of drug resistance
Surveillance	Strengths: A recording and reporting system has been established; Leprosy recording and reporting staff for routine national data validation and initiating several recording systems such as SIKK (*Sistem Informasi Kusta dan Kusta Kontak*/Leprosy and Contact Information System), SIPK (*Sistem Informasi Pencegahan Kusta*/Leprosy Prevention Information System) (already has a decree from the Ministry) has been formed; Well-documented data cohort (soft file). Opportunities: A national e-learning basic platform for capacity building of health workers has been established; OYPMK organizations and civil society organizations can be involved in leprosy prevention and control; Use of village funds and BOK for accommodation costs for leprosy cadres, financing of cadres; Digital transformation (SITASIA, e-learning) is being developed by the Ministry of Health.	Weaknesses: Implementing active case findings is not intensive Leprosy reporting systems in hospitals and private health services are not yet connected with the national data system Reporting system mechanisms for mobility patients are not available Evaluating case-finding activities are not yet implemented regularly. Threats: Lack of awareness of self-reporting or reporting suspected leprosy among the community.
Stigma	Strengths: The involvement of NGOs and OYPMK in reducing stigma against leprosy has been established; Government and private information broadcasting platforms and facilities for education to reduce stigma have been established; Several national programs implemented at various levels of government, including villages such as PIS-PK, PKH, and KB, can support leprosy programs. Opportunities: OYPMK organizations, civil society organizations, community leaders, religious leaders, and cultural leaders consistently support the leprosy program; Availability of various social and cultural systems that influence the community level (houses of worship, etc.); Availability of cadre funding from village funds and BOK; Indonesian society is one of the largest countries using social media in the world	Weaknesses: Health promotion media is inadequate for the community and health facilities regarding quantity, content, and distribution reach. Threats: Stigma against leprosy remains high in society and remains rejected by health workers in case detection and prevention.

### 3.2 Proposed improvements

Three proposed improvements emerged from the participants during online group discussions. The first is community participation and involvement of other sectors, such as the private sector, in leprosy control. These can include gaining community support, collaborating with multiple sectors and stakeholders, engaging the private sector (e.g., private clinics and specialist doctors), and optimizing community activities at the village level (such as Bina Desaku or Leprosy Friendly Village). The case findings can be improved by forming potential village groups for health education and developing tools for cadres to track and detect cases. Health education should involve stakeholders, religious and village leaders, the community, and other potential groups to disseminate messages and reduce stigma.

Second, the quality of the leprosy program management should be improved. These include improving drug and logistics management for MDT and SDR-PEP, especially for children affected by leprosy outside the Java region; conducting training on leprosy in all healthcare facilities; optimizing early detection, treatment, monitoring, and evaluation; utilizing the existing public-private mix (initiated by tuberculosis and non-communicable disease programs); disseminating information through social media; ensuring adequate and equitable budget allocation for case detection; and motivating health workers to provide treatment.

Finally, improvements in advocacy and policy alignment should be made, such as revising the national action plan in line with the national health system transformation agenda, supported by the social insurance policy on benefit packages. Moreover, indicators for leprosy elimination should be considered according to the local context, supported by strong advocacy to reach village leaders and the community and lobbying for sustainable funding allocations.

## 4 The national action plan: strategies and key interventions

Through a series of workshops and discussions, NAP-L 2023–2027 was produced with the aim of decreasing the number of new cases, new cases among children, and new cases with disabilities (Table 4).

**Table 4:** Indicators and target of indicators in the National Action Plan for Leprosy 2023–2027 and 2030.

Indicators	Target						Toward 2030
	2019 (baseline)	2023	2024	2025	2026	2027	
Number of new cases per year (% decrease)	17,439	15,695 (10%)	13,951 (20%)	12,207 (30%)	10,463 (40%)	8,719 (50%)	5,231 (70%)
Number of new cases in children per year (% decrease)	2,009	1,808 (10%)	1,607 (20%)	1,406 (30%)	1,004 (50%)	602 (70%)	200 (90%)
Number of new cases with grade 2 disability (% decrease)	1,121	1,008 (10%)	896 (20%)	784 (30%)	560 (50%)	336 (70%)	112 (90%)

To achieve these targets, NAP-L sets out four pillars with annual targets: (i) mobilizing various community resources (community); (ii) increasing the capacity of the healthcare system in the prevention, early detection, diagnosis, and management of leprosy in a comprehensive and quality manner (acceleration); (iii) improving integration and coordination with stakeholders and public-private healthcare providers (integration); and (iv) strengthening commitment, policy, and leprosy program management (commitment, policy, and management). Details of the strategies and key interventions are presented in Table 5.

**Table 5:** Strategies and key interventions in the National Action Plan for Leprosy Control in Indonesia.

Strategy 1. Mobilizing community resources (community)	
Key intervention 1	Empowering community health workers (*cadre*), people who have experienced leprosy (persons affected by leprosy), and their families to support outreach, prevention, and detection of leprosy.
Key intervention 2	Strengthening the role of peers in the stigma removal and improving mental well-being and fulfillment of the rights of people who have experienced leprosy.
Key intervention 3	Strengthening the role and capacity in raising awareness and resource mobilization for leprosy elimination.
Key intervention 4	Increasing the role of family and community in providing assistance to patients with leprosy and *persons affected by leprosy* in providing support to improve health social participation and access to health rehabilitation services.
Key intervention 5	Strengthening the role of *persons affected by leprosy* and people with disabilities, their communities, or organizations in mobilizing support, policies, and resources to achieve leprosy elimination and stigma removal.
Key intervention 6	Developing innovative and participatory health promotion strategies using various information channels.
Strategy 2. Increasing the capacity of the healthcare system in prevention, early detection, diagnosis, and management of leprosy in a comprehensive and quality manner (acceleration)	
Key intervention 1	Increasing primary health services, comprehensive referrals, and support in government or private health service facilities.
Key intervention 2	Involving professional organizations and academic institutions in developing clinical leadership and expertise in the field of leprosy.
Key intervention 3	Increasing early case finding of leprosy in at-risk populations through active case finding in the community (including targeting women and remote areas).
Key intervention 4	Improving contact management coverage and quality of leprosy chemoprophylaxis.
Key intervention 5	Improving the monitoring and care for current and former patients with leprosy.
Strategy 3. Improving integration and coordination with stakeholders and public-private health care providers (integration)	
Key intervention 1	Improving coordination, collaboration, and integration of leprosy elimination programs with other health programs, starting from the central, regional, and health facility levels.
Key intervention 2	Improving coordination, collaboration, and integration of key interventions in the leprosy elimination program with programs in relevant cross-sectors.
Key intervention 3	Enhancing advocacy at central and local levels to develop partnerships with the private sector, philanthropy, communities, development partners, and other multi-sector partners according to their capacity and competence.
Key intervention 4	Strengthening public and private partnerships for leprosy services.
Strategy 4. Strengthening commitment, policy, and leprosy program management (commitment, policy and management)	
Key intervention 1	Making leprosy one of the indicators of successful regional development.
Key intervention 2	Strengthening central and regional commitment to leprosy elimination through affirmative policies for resource allocation to endemic areas, especially for the underdeveloped, border, and island areas.
Key intervention 3	Harmonizing policies that support the achievement of leprosy elimination and reduction of stigma and discrimination.
Key intervention 4	Improving governance and leadership in leprosy control programs at the central and regional levels.
Key intervention 5	Strengthening leprosy control program management in provinces and districts/cities.
Key intervention 6	Improving leprosy research and its utilization to strengthen program implementation.

### 4.1 Community (strategy 1)

Strategy one emphasizes the role of patients, families, and communities as the primary focus of prevention and leprosy control programs. This strategy aims to: (i) improve the behavior of family, community, and community leaders related to prevention and leprosy control; (ii) increase access, quality, and information dissemination to educate families and the community; and (iii) optimize the role of religious and community leaders in using various media for health education.

### 4.2 Acceleration (strategy 2)

Strategy two ensures comprehensive healthcare services for leprosy from prevention to rehabilitation. This strategy has three specific objectives: (i) to improve access and quality in managing patients with leprosy at the PHC and hospital levels; (ii) to prevent or conduct early detection of new cases and disabilities; and (iii) to provide a positive experience for patients, families, and communities when visiting health care facilities.

### 4.3 Integration (strategy 3)

Strategy three concerns multi-stakeholder integration and collaboration at all levels, from the national to the village level. This strategy has two specific objectives: (i) to improve coordination and collaboration between leprosy control programs and other national priority health programs and cross-sectoral programs and (ii) to integrate leprosy control programs at the policy and implementation levels. The focus of integration is to create content on leprosy for health education, strengthen the integration between leprosy and other health programs, and integrate data monitoring.

### 4.4 Commitment, policy, and management (strategy 4)

Strategy four provides a support system from the national to village level to improve the resources and capacity to implement the first three strategies. This strategy includes governance, leadership, program management, and continuous improvements. The objectives of this strategy are to (i) strengthen the commitment and leadership of central, regional, and village governments through effective communication and advocacy; (ii) improve program management to acquire better resources; and (iii) increase contributions from local government funding, other health programs, and cross-sector programs, as well as resources from multiple parties.

## 5 Discussion

A national action plan is a vital component of the national health architecture that reflects the government’s commitment to building effective strategies for prevention, high-quality care, and equitable access for all. The overall goal and targets of the NAP-L were determined based on an analysis of the current situation in Indonesia, where the prevalence of leprosy is still high in endemic provinces despite the elimination status at the national level. Four strategies were formulated for NAP-L. These emphasize community mobilization; accelerating access to and quality of leprosy care services; and improving integration and coordination, supported by high commitment, policies, and management of leprosy control programs.

### 5.1 Alignment with the global and national initiatives

The long-term goal stated in the NAP-L is aligned with the GLC’s goal toward zero leprosy, that is, a triple of zero infection and disease, zero disability, zero stigma, and discrimination, with the short-term goal of elimination of leprosy defined as interruption of transmission. Similarly, the targets in Indonesia adopted the medium goal of the GLC, that is, to reduce 70% of new cases, 90% of new cases in children, and 90% of new cases of grade two disabilities by 2030 from reported cases in 2019 (21).

The dynamics of the discussions to determine the goals and targets of leprosy control reflect the complexity and challenges in refocusing on the goal of the national program to move from the elimination of leprosy as a public health problem to interrupting transmission toward zero leprosy. In 2000, Indonesia declared the elimination of leprosy at the national level, with elimination defined as a reduction in prevalence below 1 per 10,000 (22, 33). This led to the impression that the disease had disappeared and no longer required resources (34). At the provincial level, if a similar understanding is perceived by the program staff and stakeholders, resource allocation for leprosy control is only required for the six provinces that have not achieved elimination. This deviates from the fact that even a single case of leprosy presents a public health problem for local health officials (35). A recent evaluation of leprosy control in Indonesia (2023) highlighted leprosy as a major public health problem, since 491 of 514 (95.5%) districts reported new cases of leprosy (36).

Thus, the development of NAP-L in Indonesia is critical for responding to the country’s situation. It also fulfilled the first strategic pillar of the GLC, that is, to implement integrated, country-owned, zero-leprosy roadmaps in all endemic countries. While the remaining strategies in the GLC were adopted in the national strategy, the sequence of the NAP-L strategies differed. The document emphasizes the patient-community-centered perspective with a chain of quality improvement starting from the patient-families-community, the microsystem where patient families interact with healthcare providers, the macro system at the healthcare organization level, and finally, the environment that affects healthcare organizations (31).

Compared with the NAP-L from other countries (e.g., India, Tanzania, Nepal, Timor Leste), the process of developing the national action plan was generally identical, starting from the situation analysis, and challenges in the implementation of the previous action plan. Gaps were then identified from the epidemiological, patient-centered, and healthcare system perspectives. Finally, the proposed strategies and interventions were outlined. Similar strategies were identified, such as strengthening commitment and political support (India, Nepal dan Timor Leste); increasing the healthcare system’s capacity, and community-based interventions to reduce stigma (all four countries). In contrast, the four countries have not explicitly stated integration and coordination strategies in their national leprosy strategy, unlike Indonesia (37–40).

The first strategy of NAP-L (i.e., community mobilization) highlights the importance of putting patients’ and communities’ perspectives first. Previous studies have shown that leprosy remains stigmatized in the Indonesian community (41, 42), partly because of a lack of knowledge about leprosy and poor access to information (43). Leprosy is often believed to be a punishment from God, easily transmitted, and persons affected by leprosy (especially those with disabilities) must, therefore, be excluded from society (44). Although significant progress has been made in addressing stigma and discrimination (such as the Leprosy Friendly Village approach in North Sulawesi, Indonesia), strong stigma in the community can seriously hinder the leprosy control program by preventing or delaying access to medical care (22, 45). This patient-family-community strategy corresponds well with the last strategic pillar of the GLC in combating stigma and ensuring the human rights of patients (21).

In pragmatic ways, community-driven initiatives have effectively enhanced awareness of leprosy, reduced stigma, increased early detection of cases, improved treatment adherence, and promoted community rehabilitation (46–49). Reports from countries where leprosy is endemic (e.g., Brazil, India, Nigeria, Bolivia) highlight the importance of community-led efforts in developing culturally sensitive and inclusive approaches that address inequalities in leprosy prevention and treatment access (46, 50–55). These initiatives also empower local communities to determine the most suitable strategies for implementing leprosy programs that fit their contexts and can drive cost-effectiveness (i.e., volunteers and community health workers) (46, 56–58). Like any health policy change or community-focused intervention, the National Action Plan on Leprosy’s (NAP-L) success depends heavily on adhering to the proposed interventions.

The second strategy of the NAP-L accelerates access to and quality of leprosy case detection, treatment, and chemoprophylaxis, in line with the second and third GLC strategies focusing on integrated active case detection and prevention, managing complications, and preventing new disabilities. In the short-term, the most promising innovation for reducing the transmission of *M. leprae* is enhanced case detection and MDT treatment for those diagnosed with leprosy, along with chemoprophylaxis for their contacts and communities (59), which is implemented in this second strategy. If stigma is not adequately addressed, the problem of patient delay can significantly impede leprosy care regardless of the availability of effective interventions for case detection, treatment, and prevention of disability and the promising development of vaccines and new diagnostic tests (60). A study conducted in Central Java, Indonesia, showed that the mean case detection delay and patient delay were 13.0 months and 9.7 months, respectively (29).

The strategy of improving integration and coordination with stakeholders and public and private healthcare providers (strategy three) represents a crucial pillar for leprosy control in Indonesia. The importance of collaboration between leprosy control and other health programs (programmatic synergy) and multi-sectoral collaboration cannot be overstated in leprosy, which affects the physical and emotional status, social well-being, and economic welfare of persons affected by the disease and their families. Synchronized efforts should be planned and implemented in all spectra, from promotive and preventive activities to preserve household contact; advocacy with stakeholders related to leprosy prevention policies; and assisting individuals, families, and communities to play active roles in the discovery and management of patients with leprosy, the implementation of chemoprophylaxis, and research and development activities. For instance, a recent experience in Nepal highlights the feasibility and potential synergies of integrating services and training in self-care for people whose limbs are affected by leprosy or lymphatic filariasis (61).

Regarding healthcare providers, the number and spread of public and private healthcare facilities at primary care and referral hospital levels in Indonesia provide opportunities and challenges for the leprosy control program to work collaboratively. Leprosy control may take lessons learned from other disease control programs, such as HIV/AIDS and tuberculosis, which have more experience in effectively engaging diverse stakeholders and public-private providers (62). Thus, with persistent concerns for promoting equitable access and ensuring the effectiveness of leprosy care while sustaining community-led participation, the integration strategy in the NAP-L consolidates not only the program’s dimensions but also reorients the practice of healthcare toward a more integrated approach and collaborative work within health systems (27, 63).

These three strategies in the NAP-L can only be implemented successfully if supported by strong commitment, policy, and management (strategy four). This strategy is linked to the broader goal of transforming the national health system toward resilience (64). A resilient health system is critical for overcoming the high number of cases of neglected communicable diseases, delays in early detection, and access to treatment to prevent disability. As Indonesia undergoes significant reforms to enhance the accessibility, quality, and equity of healthcare services, addressing leprosy within health system-strengthening initiatives and the development of an extensive, widely skilled healthcare force is paramount to achieving comprehensive outcomes in leprosy control (65, 66). Although a policy on leprosy control exists (i.e., the Ministry of Health regulation in 2019), lack of or low resources coupled with loss of expertise are still major concerns that should be addressed in this strategy (34). Maintaining interest, resources, and expertise, and transitioning the resources and expertise of leprosy control so that they are not totally lost but gradually redirected to contribute to the remaining health problems, are the major advocacy challenges throughout the world (34).

### 5.2 The process of developing the national action plan

The content of the NAP-L and its development process generally comply with the requirements of the International Health Partnership to ensure the quality of national strategies (such as the NAP-L). The Joint Assessment of National Health Strategies and Plans (JANS) tool describes five specific sets of criteria as essential components and parameters for an excellent national strategic health plan. It consists of (i) situation analysis and programming; (ii) the development of national strategies and plans; (iii) the cost and budget of the plan to implement the relevant strategy; (iv) implementation and management arrangements; and (v) results, monitoring, and review mechanisms (67).

The process recognizes that in conducting situation analysis and identifying strategies, diverse resources and various perspectives are substantial to marshal (68). Applying the policy triangle framework, which consists of actors, context, and content (69), actors were identified from the government (cross-program and cross-sectors), non-governmental organizations, civil society organizations, academia and health providers, the private sector, persons affected by leprosy, and communities of practice at the national and subnational levels. The context analysis relied on strengths, weaknesses, opportunities, and threats (SWOT) analysis, and the content addressed the objectives and provided strategies in alignment with the GLC Global Leprosy Strategy 2021–2030 and the National Strategic Plan of the Ministry of Health Indonesia, 2020–2024 (21, 23).

Throughout the development of the NAP-L, a transparent and stepwise process existed to demonstrate the government’s commitment and obtain meaningful participation from multiple stakeholders. The results of each workshop were summarized and presented in a follow-up workshop. This enabled continuity and feedback from various stakeholders. With the roles of stakeholders clearly outlined in the NAP-L document (Supplementary 3), the process of developing the NAP-L attempted to foster the commitment and ownership of stakeholders in implementing it. Overall, the NAP-L process mirrors the preparatory process of a national strategic plan for tuberculosis (70).

The complexity of leprosy control, especially related to stigma reduction and prevention of disability, requires participation from a community of practitioners and interest groups led by the society for various purposes (71). NGOs and persons affected by leprosy organizations were involved as part of advocacy and broader outreach for individuals who may be unreachable by the government, to increase trust and support for leprosy elimination efforts among those affected by leprosy, and to develop social capital in the leprosy community. For example, Perhimpunan Mandiri Kusta (PerMaTa) operates at the national level in seven provinces in Indonesia and builds trust among its members and with the public and partners by fostering better relationships within the organization, disseminating accurate information through media channels, and establishing norms that balance the rights and responsibilities of all members. Peduli Disabilitas dan Kusta (PELITA), a consortium of 22 community-led organizations, brings together organizations, the government, and/or private entities involved in the prevention, management, and empowerment of people with disabilities and persons affected by leprosy to achieve an inclusive Indonesia free from leprosy and assist the government in human development programs related to people with disabilities and people affected by leprosy. Therefore, organized partnerships at the national and subnational levels can help ensure concerted efforts to eliminate leprosy and prevent the duplication of services. At the global level, the leprosy community has come together in a new Global Partnership for Zero Leprosy, as full partners in the larger NTD community share fruitful exchanges of insights, knowledge, and approaches (60).

### 5.3 Cross-cutting issues

Leprosy control is closely related to protecting human rights, gender, justice, and ethics. The issues of human rights, gender, and justice are relevant not only from the perspective of delayed case detection but also from the perspective of disability and its social and lifetime consequences due to leprosy. Therefore, leprosy control programs must put forward more sensitive measures and prioritization to protect vulnerable groups. The involvement of women is crucial to address the challenges of leprosy elimination and make significant changes to families and communities. Special attention should be paid to children and women to regularly promote and participate in early detection and screening, and facilitate diagnosis, care access, and leprosy treatment (72–74).

The Global NTD response further elaborates on cross-cutting issues by dedicating one strategic pillar, the cross-cutting approach, prioritizing integration across NTDs, cross-sectoral coordination, and mainstream within national health systems (75). At the global level, leprosy control is embedded within the broader NTD effort, in which shared experiences and program integration are key themes. Leprosy is now well represented in major NTD gatherings such as annual meetings of the NTD NGO Network and the Coalition for Operational Research on NTDs (46). In Indonesia, the Ministry of Health recently included leprosy in the annual report of 10 selected programs for the purpose of advocating for stakeholders and potential funders (76).

Zero-leprosy requires a paradigm shift from a disease-elimination response to an integrated health system response according to the community’s needs. Therefore, it is vital to ensure alignment with national priorities and facilitate adequate engagement and participation of other key institutions and agencies within and beyond the health sector to achieve multiple goals of NTD control (i.e., interruption of transmission, reduction of leprosy burden, stigma reduction, and prevention of disability) in attaining sustainable development goals (77).

Cross-cutting contributions and harmonization from various institutions, agencies, and disciplines should be coordinated to promote programmatic synergies. Current practices for leprosy control are mainly disease-specific (i.e., MDT, detection, prevention, and elimination). Social and health system factors such as political will and commitment, financial status, equity access, lack of data, and continuity of care are still fragmented. Thus, by harmonizing policies, such integrated cross-cutting approaches have led to an optimized impact, maximized the utilization of available resources, and ensured synergies among various institutions and stakeholders to achieve leprosy elimination targets (66, 78, 79). This approach should leverage existing collaborations with leprosy experts at academic and research institutions. For example, the Ministry of Social Affairs is responsible for formulating and implementing policies in the fields of social rehabilitation, social security, social empowerment, and social protection for leprosy sufferers, people affected by leprosy, families, and communities. Social services play an important role in supporting the policies and programs of the Ministry of Social Affairs in social rehabilitation programs for persons affected by leprosy and in carrying out education and training in the context of social welfare development efforts for persons affected by leprosy at the provincial and district/city levels. Universities, academics, researchers, and research institutions play roles in formulating, developing, and enriching research and technology to support the success of leprosy elimination programs.

### 5.4 Implications for future implementation

The NAP-L provides a strategic framework for monitoring, evaluation, and continuous quality improvement to achieve zero leprosy infections, in line with the GLS. Its effectiveness in reducing the burden of leprosy can be enhanced by translating this national action plan into a district-level action plan and prioritizing strategies and interventions to suit the local situation, followed by a rigorous monitoring and evaluation system. Thus, local government commitment and intersectoral collaboration are key to providing adequate resource allocation for implementing a local action plan.

To monitor and evaluate the processes and outcomes of the NAP-L, the indicators were aligned with the GLC indicators. Therefore, the measurements should be embedded in the management system. In addition, efforts to identify the barriers to implementing an action plan, along with alternative solutions and plans for improvement, should be well documented. Implementation research may further strengthen the systematic monitoring and evaluation of leprosy control. Specific areas such as case mapping, improving data management, monitoring and surveillance, and strengthening the health system were considered priorities in implementation research for zero leprosy. This research agenda can be further developed from global or SEARO research priorities to identify research priorities for Indonesia (80, 81). Prioritization should be driven to achieve zero leprosy and not limited to the six provinces that have not yet been eliminated. Through appropriate dissemination and communication of research findings, while maintaining public attention, the performance and quality of the leprosy control program can be improved toward zero leprosy (80, 82).

Adequate resources at the operational level, where leprosy control is implemented, should be ensured by understanding budget requirements and exploring multiple sources of funding. Applying the cost-per-activity approach for each strategy and key intervention, the total budget required for implementing the NAP-L in Indonesia in 2023–2027 is estimated to exceed two million US dollars. The increased capacity of the healthcare system for the prevention, early detection, diagnosis, and management of leprosy in a comprehensive and qualitative manner (strategy two) requires the largest estimated budget allocation, followed by resources to strengthen commitment, policies, and leprosy control program management (Strategy four).

The availability of financial resources is challenged by a reliance on resources allocated by the central government budget and low contributions from grants, foreign aid, the private sector, and philanthropy for leprosy control. The transition to a new government in 2024 and the year 2025 is a transitional year between the current national long-term development plan (2005–2025) and the new national long-term plan (2025–2045) are two additional, high agendas at the national level that can result in health programs.

The NAP-L is a nationally coordinated effort to address leprosy in Indonesia. Considering the complexity, lack of political will, unmet needs, and stigma associated with the burden of leprosy, a comprehensive approach is required. Informed by extensive stakeholder participation and input, the NAP-L has made significant efforts to address present and future challenges. It is outlined as a collaborative effort through community participation, accelerating healthcare system capacity, integration, coordination, leadership, and management.

Three significant potential barriers should be considered. First, the imbalance of health systems capacity within the province or district levels. Implementing a national action plan for leprosy by provincial and district governments presents both an opportunity and a challenge. It requires careful consideration when adopted at the subnational level, particularly in the context of health systems decentralization. Not all provinces and districts possess the necessary capacity to adopt the NAP-L. To address implementation and adoption barriers, the central government should provide technical assistance and strong political commitment. These strategies aim to support local governments in developing feasible, scalable, affordable, and cost-effective interventions. By doing so, health policies can promote regulatory, legislative, and inter-sectoral collaborations to address leprosy with a locally sensitive approach.

Second, limited resources and inadequate funding significantly impair NAP-L implementation and its sustainability. These factors cause unavailable or insufficient treatments and logistics, patient loss to follow-up, inadequate case detection, delayed treatment, and prolonged stigma due to a lack of health promotion. The shortage of staff and low capacity for testing and treatment impair healthcare workers’ ability to comply with recommendations that worsen the quality of care for the patient. To address these issues, we have provided a detailed description of tasks and responsibilities among stakeholder and partnership institutions. Also, to provide quality care, essential healthcare services should be maintained with strengthened technical support, and collaboration with NGOs and community organizations.

Third, the current government’s budget efficiency policy may lead to reduced case-finding activities, as well as recording, reporting, therapy, and dissemination aimed at reducing stigma. As a consequence, the burden of leprosy may be increasing, and local governments may struggle to implement the action plans. To address this issue, the involvement of philanthropies to provide funding for leprosy programs in endemic areas should be explored.

The NAP-L highlights essential elements, such as the participation of the community and persons affected by leprosy, integrated care, political commitment, and sustainable mechanisms to drive implementation. These processes and approaches for the development of NAP-L can be adopted to other disease programs, particularly for NTDs endemic to the region.

### 5.5 Strengths and weaknesses

The complex nature of leprosy requires thorough consultation with multiple stakeholders. In total, 55 institutions representing stakeholders from different levels (national to community) were engaged throughout the process. Deliberate efforts were made to involve a wide range of public-private stakeholders and partners (including leprosy control programs, other health programs, and non-health sectors), non-government organizations, professional associations, public-private health care providers, and, most importantly, persons affected by leprosy. Multistakeholder engagement is critical for soundness and inclusiveness in developing national strategies (70).

Similarly, participation in other health programs at the national level during development is essential, particularly for integration strategies. This was more challenging during the development process because of competing activities despite the Ministry of Health’s leadership continuously sensitizing and strengthening the importance of the NAP-L to a wide range of stakeholders. During workshops, technical obstacles often occurred owing to unstable Internet network connections, particularly in endemic areas in the eastern part of Indonesia. Thus, stakeholders from representative areas were unable to fully participate in the NAP-L development. In addition, online discussions often limit the dynamic interactions among participants. To minimize these issues, all online discussions were moderated by two facilitators (i.e., lead facilitator and co-facilitator). The lead facilitator was responsible for setting the context, driving the discussion, and engaging the participants in an interactive discussion. A co-facilitator was responsible for admitting and organizing participants in the virtual waiting room, muting participants who may unintentionally distract others, and resolving technical problems during the discussion. The core team provided an interactive screenboard to present the keywords of the responses and participant feedback. Participants were also informed to disconnect other devices from the Wi-Fi and avoid outside distractions.

For each strategy, the key interventions were identified and agreed upon during the workshops. Broader evidence of effective program interventions was not sought, and consensus methods were not applied in the development of the NAP-L. Although these would ensure the effectiveness of interventions included in the national strategy, neither the JANS nor the WHO toolkit to develop the national strategic plan for TB put sufficient emphasis on its importance (67, 70). Due to the COVID-19 pandemic, discussions and workshops have been held virtually. To elaborate on the critical consensus from the workshops and unstable access to Internet networks, the NAP-L facilitator team provided guidelines for a virtual discussion that contained procedures, audio recordings, notetaking, transcripts, and mind map tools to facilitate the discussion process. To ensure inclusivity and minimize social rejection, individuals affected by leprosy participated in all the serial workshops.

Efforts to reduce the burden of leprosy in Indonesia would depend on the existing healthcare system, its network, and available healthcare workers. Transformation of the primary care system in Indonesia is expected to strengthen the linkages between public and private care facilities at the primary care level, as well as linkages to the community to enable comprehensive care required for leprosy care. However, challenges remain regarding the complexity of a decentralized health system in the implementation of leprosy control programs. Inadequate resource allocation and stakeholder commitment, logistics and drug stockout, low levels of competence in diagnosis and treatment among healthcare workers, and social rejection due to stigma vary (29, 83, 84).

## 6 Conclusion

The National Action Plan for Leprosy (NAP-L) 2023–2027 provides a robust framework for achieving zero leprosy in Indonesia by 2030. By setting clear targets, the NAP-L addresses the critical gaps in leprosy control. To achieve these targets, four main pillars–community, acceleration, integration, and commitment-policy-management, along with the 21 key interventions, should be implemented and further adjusted by local governments to reflect local challenges, prioritization, and available resources. The NAP-L provides a roadmap with crosscutting approaches to reduce stigmatization, improve early diagnosis, raise awareness among health professionals and communities, and increase access to rehabilitation services. Strong and aligned regulations at the central and local government levels are required to ensure the implementation of the NAP-L, particularly concerning resource allocation. Therefore, an effective strategy for advocacy and communication is a requirement for the NAP-L to be successfully adopted at the local government level, particularly in high-burden areas. The NAP-L is expected to be a guideline for developing provincial and district action plans to eliminate leprosy. Further adaptation of the national action plan to best suit local situations, challenges, and priority interventions is crucial, considering the diverse settings and availability of resources at the provincial and district levels in Indonesia.

The structure and strategies outlined in the NAP-L can be adopted as a model for other NTD programs in LMICs, demonstrating the importance of inclusive, evidence-based policymaking in achieving health equity. The NAP-L development process and proposed strategies are relevant to global public health policy in the following aspects: (1) demonstrating a participatory policy-making process from various sectors, ensuring inclusivity and ownership; (2) providing a replicable model for integrating NTD control into decentralized health systems, particularly in low- and middle-income countries (LMICs); (3) highlighting the importance of linking national policies to global strategies, such as the GLS 2021–2030 and the SDG, particularly SDG 3 (Good Health and Well-being) and SDG 10 (Reduced Inequalities); and (4) incorporating implementation research to strengthen monitoring, evaluation, and adaptation of strategies, ensuring that evidence-based practices are integrated into policy and program delivery. Therefore, this contributes not only to national health priorities but also to global public health literature.
