# Supplementary material for: Participatory development of Indonesia’s national action plan for zero leprosy: strategies and interventions
Source: Front Public Health. 2025 Apr 10;13:1453470. doi: 10.3389/fpubh.2025.1453470 (PMC12018332; doi:10.3389/fpubh.2025.1453470)
Supplement: Supplementary file 1 [file Table_1.docx]

***Supplementary Materials***

**Supplement 1**. Summary of participants by level, institution, type, and number of people engaged

| Level | Institutions | Number of Participants |
| --- | --- | --- |
| National and sub-national | The Executive Office of the President of the Republic of Indonesia  Commissioner of National Disability Commission  WHO Indonesia  Ministry of Health: Directorate of Communicable Disease Control; Directorate of Mental Health; Directorate of Public Health Management; Directorate of Health Promotion and Communicable Diseases; Directorate of Non-communicable Disease Prevention;  Provincial Health Offices: Nusa Tenggara Timur, Jawa Barat, Papua, Papua Barat, Maluku, Sulawesi Tengah, Sulawesi Utara, Kalimantan Utara, Jawa Tengah, Gorontalo, Maluku, Maluku Utara, Jawa Timur, Sumatera Utara | 39 |
| District | District Health Offices: Bandung, Kupang, Aceh, Indramayu, Subang, Kuningan | 6 |
| Health care facilities | Public health center (Balkesmas);  National referral hospitals;  District hospitals; Private hospitals;  Primary Health Centers (Puskesmas); | 14 |
| Academia, training center | Universities/Research centers, Centre for Environmental Health Engineering and Disease Control | 4 |
| NGO and community-led organizations | NLR Indonesia;  Satu Jalan Bersama Foundation;  Gerakan Peduli Disabilitas dan Lepra Indonesia;  Yayasan PerDIK;  PerMaTa Bulukumba;  Konsorsium PELITA;  Sentra Terpadu Pangudi Luhur Bekasi;  FKDC;  PerMaTa Indonesia;  FORMASI Disabilitas;  Dedikasi Tjipta Indonesia Foundation;  DSM | 15 |
| Total number of participants | | 78 |

**Supplement 2**. Characteristics of participants in the online group discussions (n=78)

| OGD | Group of Stakeholders | Sub-groups | Facilitators | No. of participants |
| --- | --- | --- | --- | --- |
| 1 | National Leprosy Control Program | Ministry of Health, c.q. the leprosy control program  NLR Indonesia | AU & AF | 11 |
| 2 | Leprosy control program managers | Provincial health offices | AU & AF | 17 |
| 3 | Leprosy control program managers | District health offices | PHS & AJAYR | 13 |
| 4 | Healthcare providers | Specialist and general physicians working at hospitals, primary care centers, and clinics  Professional associations | AJAYR & PHS | 21 |
| 5 | Academia, NGOs, and community-led organizations | Researchers and lecturers  Organizations for persons affected by leprosy  Disabled person organizations | AJAYR & PHS | 16 |

**Supplement 3.** Workshop themes, objectives, and outputs to develop a national action plan for leprosy in Indonesia

| Workshop themes | Objectives | Duration | Outputs | No. of participants attended |
| --- | --- | --- | --- | --- |
| Workshop 1: Situation analysis | Assess the epidemiological situation of leprosy in Indonesia;  Provide an overview of the current situation in leprosy control; | 5.5 hours | Draft of the situation analysis, consisting of epidemiological burden, diagnostic and case management, prevention and control including chemoprophylaxis, surveillance, human resources, financing, information system, and research and innovation | 40 |
| Workshop 2: Stakeholder analysis | Identify and map different stakeholders at different levels and across multiple sectors;  Identify the interests and importance of different stakeholders at various levels;  Identify key strategies and actions to engage different stakeholders in the implementation of the NAP-L | 3 hours | List of stakeholders, including roles and responsibilities in leprosy control  Map of stakeholders, power, and interest in leprosy control | 41 |
| Workshop 3: Strategic issues | Identify strategic issues or challenges in leprosy control;  Identify introduced opportunities and threats (SWOT analysis) | 3 hours | SWOT analysis and strategic issues  Draft objectives, targets, and indicators | 34 |
| Workshop 4: Strategies | Formulate goals and objectives using the SMART criteria (Specific, Measurable, Achievable, Relevant, and Time-bound criteria);  Identify strategies that are potentially effective for leprosy control;  Identify available resources to achieve the targeted goals. | 3 hours | Draft strategies for leprosy elimination for NAP-L, including Objectives and indicators for each strategy | 34 |
| Workshop 5a: Key interventions | Develop key interventions and activities for strategies 1-2; | 4 hours | Draft of key interventions and activities for strategies 1-2 | 75 |
| Workshop 5b: Key interventions | Develop key interventions and activities for strategies 3-4; | 4 hours | Draft of critical interventions and activities for strategies 3-4 | 66 |
| Workshop 6: Implementation strategies | Identify approaches for implementing the main strategies and key interventions, including objectives, activities, locus, targets, population, and or service targets;  Develop monitoring and evaluation mechanisms for the implementations of NAP-L;  Identify key roles and responsibilities for implementing key strategies and interventions. | 6 hours | Draft monitoring and evaluation framework  Draft implementation strategies | 47 |
| Workshop 7: Program Budgeting | Develop budget plans for leprosy control;  Identify the unit cost of each activity according to the objectives, key interventions, and strategies in the NAP-L | 3.5 hours | Costing document | 44 |
| Workshop 8: Financing | Identify the resources available from each stakeholder at different levels;  Identify the availability of the existing budget from key stakeholders | 2.5 hours | Mapping of available resources  Financial plan for implementation | 44 |
